# Ischemia–reperfusion injury after spinal cord decompressive surgery—An in vivo rat model

**DOI:** 10.1002/ame2.12485

**Published:** 2024-09-03

**Authors:** Boyu Zhang, Zhefeng Jin, Pengren Luo, He Yin, Xin Chen, Bowen Yang, Xiaokuan Qin, LiGuo Zhu, Bo Xu, Guoliang Ma, Dian Zhang

**Affiliations:** ^1^ Sports medicine department 3 Wangjing Hospital Affiliated to China Academy of Chinese Medical Sciences Beijing China; ^2^ Spine Department 2 Wangjing Hospital Affiliated to China Academy of Chinese Medical Sciences Beijing China

**Keywords:** 8‐oxoG DNA, degenerative cervical myelopathy, spinal cord ischemia–reperfusion injury, surgical decompression

## Abstract

**Background:**

Although decompression surgery is the optimal treatment for patients with severe degenerative cervical myelopathy (DCM), some individuals experience no improvement or even a decline in neurological function after surgery, with spinal cord ischemia–reperfusion injury (SCII) identified as the primary cause. Spinal cord compression results in local ischemia and blood perfusion following decompression is fundamental to SCII. However, owing to inadequate perioperative blood flow monitoring, direct evidence regarding the occurrence of SCII after decompression is lacking. The objective of this study was to establish a suitable animal model for investigating the underlying mechanism of spinal cord ischemia–reperfusion injury following decompression surgery for degenerative cervical myelopathy (DCM) and to elucidate alterations in neurological function and local blood flow within the spinal cord before and after decompression.

**Methods:**

Twenty‐four Sprague–Dawley rats were allocated to three groups: the DCM group (cervical compression group, with implanted compression material in the spinal canal, *n* = 8), the DCM‐D group (cervical decompression group, with removal of compression material from the spinal canal 4 weeks after implantation, *n* = 8), and the SHAM group (sham operation, *n* = 8). Von Frey test, forepaw grip strength, and gait were assessed within 4 weeks post‐implantation. Spinal cord compression was evaluated using magnetic resonance imaging. Local blood flow in the spinal cord was monitored during the perioperative decompression. The rats were sacrificed 1 week after decompression to observe morphological changes in the compressed or decompressed segments of the spinal cord. Additionally, NeuN expression and the oxidative damage marker 8‐oxoG DNA were analyzed.

**Results:**

Following spinal cord compression, abnormal mechanical pain worsened, and a decrease in forepaw grip strength was observed within 1–4 weeks. Upon decompression, the abnormal mechanical pain subsided, and forepaw grip strength was restored; however, neither reached the level of the sham operation group. Decompression leads to an increase in the local blood flow, indicating improved perfusion of the spinal cord. The number of NeuN‐positive cells in the spinal cord of rats in the DCM‐D group exceeded that in the DCM group but remained lower than that in the SHAM group. Notably, a higher level of 8‐oxoG DNA expression was observed, suggesting oxidative stress following spinal cord decompression.

**Conclusion:**

This model is deemed suitable for analyzing the underlying mechanism of SCII following decompressive cervical laminectomy, as we posit that the obtained results are comparable to the clinical progression of degenerative cervical myelopathy (DCM) post‐decompression and exhibit analogous neurological alterations. Notably, this model revealed ischemic reperfusion in the spinal cord after decompression, concomitant with oxidative damage, which plausibly underlies the neurological deterioration observed after decompression.

## Graphical abstract

The diagram reflects the general process and focus of our research. This study focused on ischemia–reperfusion injury in the perioperative period of degenerative cervical spondylosis (DCM) in rats. We implanted the compression material into the cervical spinal canal of rats and decompressed it by C6 right hemilaminectomy after 4 weeks of compression and removal of the compression material. Behavioral tests were performed on the rats after the completion of the compression procedure and after the completion of the decompression procedure. Laser speckle contrast imaging was used to observe the blood flow changes in the compressed cervical spinal cord during decompression surgery. One week after the decompression surgery was completed, the spinal cord specimens were taken for pathological analysis.

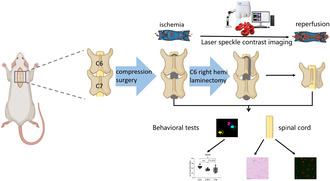

## 1 INTRODUCTION

Degenerative cervical spondylosis is a common non‐traumatic cervical spondylotic myelopathy. It includes acquired spinal stenosis secondary to osteoarthritis degeneration (such as cervical spondylosis) or distortion of the spinal ligaments (such as ossification of the posterior longitudinal ligament). Degenerative cervical myelopathy (DCM) leads to neurological dysfunction, which clinically manifests as decreased mobility and strength of the affected limbs, gait disturbances, and hyperalgesia. There is a lack of high‐quality epidemiological studies on degenerative cervical spondylosis due to the difficulty in measuring the number of mild cases. The incidence of hospitalization for this disease is 7.88 per 10^5^ persons in the United States.^1^, ^2^ In recent years, the incidence of this disease is gradually showing a trend of youthfulness.^3^ For patients with severe spinal cord compression and symptoms, decompression surgery is the first choice of clinical treatment, and is effective in preventing disease progression and improving neurological function.^4^

However, a study has reported that, following decompression surgery, 11.6% of patients experienced early postoperative neurological deterioration, and at follow‐up, this percentage increased to 17.9%.^5^ The primary cause of this neurological decline is SCII, which has been associated with various pathophysiological conditions including intraspinal surgery and thoracoabdominal aortic aneurysm repair procedures.^6^, ^7^ Spinal cord injury caused by DCM is characterized by chronic and progressive compression of the spinal cord, with chronic ischemia being one of the primary underlying mechanisms.^8^ Restoration of the blood supply to the spinal cord following decompression involves an ischemia–reperfusion process. SCII and its subsequent secondary pathophysiological reactions can result in either no improvement or exacerbation of neurological impairment in patients. Oxidative stress is the main pathological process in SCII. After perfusion with a large amount of oxygen‐carrying blood, oxygen free radicals cause continuous damage to neurons. At the same time, various pathological processes occur, including an inflammatory response, neuronal apoptosis, autophagy, and astrogliosis.^9^ 8‐Hydroxyguanine (8‐oxoG DNA) is a DNA adduct that results from elevated levels of reactive oxygen species (ROS) and serves as a significant marker for oxidative stress‐induced damage. This biomarker provides an objective measure for assessing the occurrence of spinal cord injury.^10^ There is insufficient evidence of perioperative SCII in degenerative cervical myelopathy (DCM), primarily because of the absence of appropriate animal models.

Currently, there are few reports of studies investigating animal models of SCII during the perioperative phase of decompression for DCM. In their study, Spyridon et al.^9^ developed a rat model involving spinal cord compression through the implantation of aromatic polyether materials beneath the C5–C6 lamina followed by laminectomy for decompression. However, this model lacks internal fixation, leading to compromised stability within the rat cervical spine. In our study, polyvinyl alcohol acrylamide interpenetrating network hydrogel was used as compression material. This material exhibits the desirable characteristic of uniform expansion upon water absorption while posing no chemical harm to the body. The compression material was surgically implanted into the spinal canal of rats to establish a rat model of spinal cord compression, followed by its removal via unilateral laminectomy to achieve decompression of the spinal cord. In this study, we successfully established a rat model of spinal cord decompression and conducted comprehensive assessments of behavioral outcomes, blood flow dynamics, and oxidative damage. Our findings demonstrated the occurrence of reperfusion‐induced oxidative damage following spinal cord decompression.

## 2 METHODS

### 2.1 Study design

Twenty‐four female Sprague–Dawley rats, aged 8 weeks and weighing 200–250 g, were used in this study. The rats were randomly allocated to three groups: DCM (*n* = 8), DCM‐D (*n* = 8), and sham (*n* = 8). After 4 weeks of compression, the animals in the DCM‐D group underwent decompression surgery. All animals were managed in a blinded manner throughout the experiment. After the experimental period, all rats were euthanized by an intraperitoneal injection of an overdose of sodium pentobarbital. All procedures were approved by the Institutional Animal Care and Use Committee of our Institute (approval number: BJLongan‐L‐L‐00015).

### 2.2 Compression materials

**FIGURE 1:**
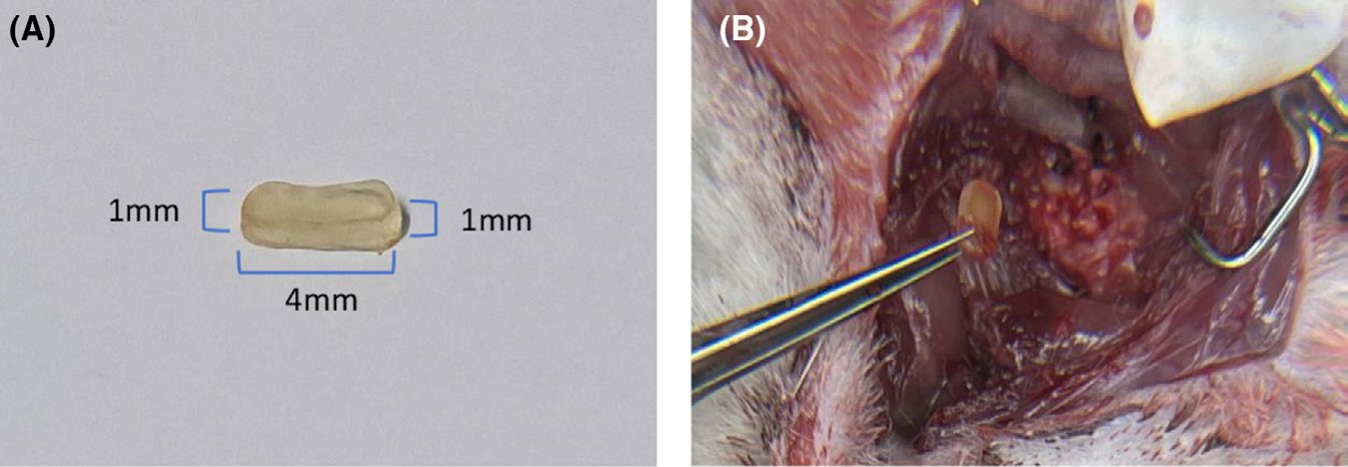
(A) Physical drawing of the material. (B) Image of material removed during decompression surgery.

A compression material composed of polyvinyl alcohol‐acrylamide interpenetrating network hydrogel (obtained from the chemical laboratory of Beijing Normal University) was used in this study. After conducting a preliminary investigation, an aluminum alloy hollow knife die was manufactured for the purpose of cutting the material. In order to mitigate potential damage or injury to the spinal cord resulting from scratching or contusion caused by the edges and angles of the resulting cubes, the ends of the cutter mold were shaped into semicircles. The final cut produces a material measuring 4 mm in length, 1 mm in width, and 1 mm in thickness. A physical depiction of the material is presented in Figure 1. To precisely position and maintain compression of the material within the spinal canal of rats, a non‐absorbable suture (5–0) measuring approximately 2 cm in length was threaded through one end and securely tied, with the fixed end positioned approximately 0.2 mm away from the edge of the material. Notably, because this procedure was conducted after in vitro expansion, it did not compromise the compressive effect exerted by the material on the rat spinal cord.

### 2.3 Spinal cord compression surgery

All procedures were performed under anesthesia with 3% sodium pentobarbital. The surgical site was shaved and sterilized with 70% ethanol. A midline incision was made in the C4‐T2 region. Following resection of the ligamentum flavum in the C6–C7 intervertebral space, a compression material was implanted beneath the C7 lamina within the same intervertebral space. The suture end of the material was positioned within the intervertebral space, and the remaining portion was placed below the C7 lamina. To ensure an unrestricted cervical range of motion in rats, a knot securely fastened the suture over the thoracic 2 spinous process. In the sham‐operated group, the compression material was removed 30 s after implantation. Subsequently, multiple tissue layers were meticulously closed to complete each procedure.

### 2.4 Decompression surgery (C6 right hemilaminectomy)

**FIGURE 2:**
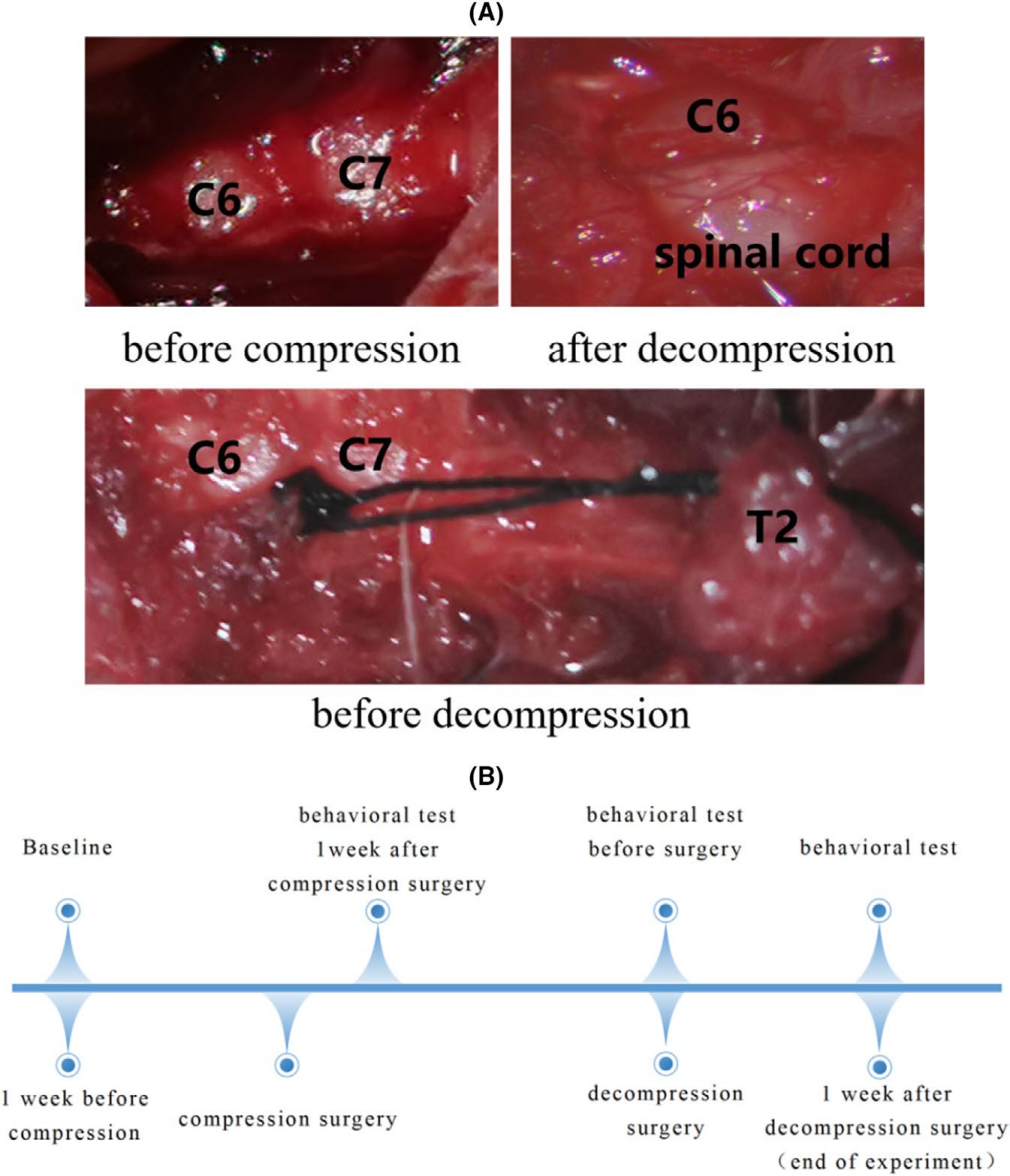
(A) Intraoperative images of compression and decompression procedures; (B) experimental cycle and behavior testing regime.

Four weeks after compression (i.e., after material implantation), the rats underwent decompression surgery. A midline incision was made in the original area to expose the C6 and C7 layers, followed by the removal of the right C6 vertebrae using bone gnawing pliers. Subsequently, the compression material was carefully clamped using ophthalmic forceps and gradually extracted to complete the decompression procedure, followed by multilayer tissue closure. In the compression group, reinsertion of the material occurred 30 s after removal. Conversely, in the sham‐operated group, laminae of identical sizes and positions were excised without intervention. (Figure 2).

### 2.5 Von Frey test

The rats in each experimental group were positioned on a shelf enclosed by barbed wire, covered with a transparent container, and subjected to testing after an acclimation period of 20–30 min. Following adaptation to the environment, the lateral plantar region of the left hind foot was stimulated using von Frey filaments of varying stiffness (ranging from 0.16 to 26 g) to assess paw withdrawal response. Fourteen touch‐tested filaments were employed, and each was meticulously calibrated to obtain consistent results. Notably, probing was conducted exclusively when all four paws of the animal contacted the floor surface. Mechanical pain threshold response (PWT) was defined as the minimum gram force exerted by a filament that elicited three consecutive stimulations on the forelimbs of each rat. Three measurements were taken per animal and averaged.

### 2.6 Grip strength test

Grip strength tests assess motor function by recording the force exerted by forelimbs. The grip strength test was divided into peak and average grip strength. During the experiment, the animal was carefully positioned to grip the metal grate with its two front paws. Subsequently, the animal's tail was gently held and pulled until the grid was released. This action stretches the spine and is, therefore, considered a measure of axial discomfort.^11^ Force data were sampled and recorded for 20 s using a YILIDA‐DS2 series push‐pull force gauge system (Shanghai Pusin Instrument Technology Co.), and the peak and average forces were calculated by analyzing the recorded load curves. This grip strength testing procedure was repeated three times, and after each trial, the rats rested for 10 min in their cages. The results of the three trials were averaged at each time point for the statistical analysis. The grip strength of all rats was normalized by dividing the grip strength by body weight at the current time point. To further explore the changes in grip strength during the experiment, the grip strengths of rats in the decompression group before compression, and after 1 week of compression, 4 weeks of compression, and 1 week of decompression were compared.

### 2.7 Gait analysis

The CatWalk XT 10.6 system (Netherlands, Noldus, Model Catwalk XT) was used to assess locomotion in animals with spinal cord compression and decompression, as previously described. Stride length, swing speed, and standing time of the forepaws and hindlimbs were analyzed 1 week before and after decompression. Full gait patterns were obtained by considering only three compliance cycles of running, in which the maximum speed varied by less than 50% per run and the mean speed did not differ significantly between runs. No food restriction or reward was used to motivate rats to perform the task.

### 2.8 Spinal cord regional blood flow testing

Measurement of regional spinal cord blood flow was conducted using Laser Speckle Contrast Imaging/LSCI (RWD Life Science Co., Ltd, China). First, the rats (*n* = 4, 4 in the DCM‐D group) were fixed in the prone position. One sham‐operated group rat was used for testing, and the remaining rats underwent decompression surgery. During decompression surgery, the subcutaneous fascia is separated, exposing the C6‐7 lamina and exposed, and the right lamina at C6 was uncovered to expose the spinal cord for the measurement of spinal blood flow. The rats were then subjected to spinal cord decompression. Blood flow in each rat's spinal cord was measured before, 5 min, and 10 min after decompression. The exposed spinal cord was defined as the region of interest (ROI) area before filming, and an ROI area of the same size was used for each rat at different time points. Three consecutive blood flow images were taken at each detection point to exclude the influence of heartbeat and respiration on the blood flow. The ratio of perfusion volume before decompression to 5, 5–10, and 10 min after decompression was calculated and expressed as a percentage of the blood perfusion rate during the two time periods.

### 2.9 Hematoxylin and eosin staining

Three rats in each group were anesthetized with sodium pentobarbital, to induce a deep coma, and then underwent cardiac perfusion with 4% paraformaldehyde (Sigma‐Aldrich, St Louis, MO, USA) solution and PBS (phosphate buffer saline). The spinal cord (centered on the compressed C6‐7 segments) was subsequently extracted and fixed overnight in 4% paraformaldehyde solution with 10% sucrose added. Tissue samples were obtained in 3‐mm‐thick sections using standard techniques and then placed in embedding molds. Subsequently, the spinal cord tissue underwent staining following gradient ethanol dehydration, clearing, wax dipping, embedding, sectioning, and baking processes. It was rinsed with distilled water for 1 min before being heated for 15 min and stained with Hematoxylin for 3 min. The sections were then briefly soaked in 1% hydrochloric acid ethanol for 1 s followed by rinsing with 2% ammonia for another second to restore the blue color; finally they were stained with Eosin for 5 min. The slices were dehydrated twice with anhydrous ethanol before being dried and sealed using neutral adhesive.

### 2.10 Immunofluorescence

2.10.1

The fixed tissue was prepared with 20%–25%–30% sucrose (three times gradient precipitation), harvested according to the requirements, and embedded with OCT until cut. Unfixed tissue was directly harvested and embedded in OCT until it was cut.

### 2.11 Sectioning and hydration control

Frozen sections 10 μm thick were made and the tissue was glued onto a slide. The cells were fixed in 4% paraformaldehyde for 30 min, shaken, and washed three times in PBS (PH7.4) on a decolorization shaker for 5 min each.

### 2.12 Frozen fluorescent double staining

After washing in distilled water, the slides were rinsed three times with PBST, shaking for 5 min each time, before being drip‐fed with membrane breaking solution for 10 min. After rinsing three times with PBST, shaking for 5 min each time, bovine serum albumin V working solution was dripped onto the slides, which were then incubated at room temperature for 30 min. The slides were then drained and the appropriate proportion of primary antibody diluted in antibody diluent was added drop by drop. The slides were then incubated at 4°C overnight, then rinsed 3 times with PBS while shaking for 5 min each time, and the corresponding fluorescent secondary antibody was added to the incubator at 37°C for 30 min (in the dark). The slides were then rinsed three times with PBS, shaking for 3 min each time (in the dark), and the nuclei were stained with DAPI for 10 min (in the dark), and then rinsed three times with PBS, shaking for 5 min each time. Anti‐quench water‐soluble sealer was applied, and the slides underwent fluorescence scanning.

The following antibodies were used. Primary antibody: anti‐8‐oxoG DNA; anti‐NeuN; dilution ratio: 1:300. Secondary antibody: goat anti‐mouse IgG H&L (Alexa Fluor® 488); goat anti‐rabbit IgG H&L (Alexa Fluor® 594); dilution ratio: 1:500. The number of NeuNs and the number of NeuNs containing 8‐oxoG DNA within a 100‐μm circular segment of the dorsal horn of the rat spinal cord were counted, and the proportion of 8‐oxoG DNA expression in NeuNs was calculated.

### 2.13 Magnetic resonance imaging

In the compression group, quantitative evaluation of spinal cord compression was performed using magnetic resonance imaging (MRI) 3 weeks postoperatively. Rats were anesthetized with 3% pentobarbital sodium and positioned in a prone orientation within a linear volume resonator for RF transmission. A flat receiving‐coil with a diameter of 30 mm was fixed directly above the neck. T2‐weighted images were acquired in the sagittal plane within the implant area, to assess the degree of spinal cord compression. Sagittal T2 fat‐suppression images were acquired using the following parameters: TR‐2900, TE‐110, FOV 120 mm, and slice thickness 2 mm. Sagittal T2 images were obtained using a TR‐3200, TE‐76, FOV of 120 mm, and a slice thickness of 2 mm. Transverse images were acquired with a TR‐400, TE‐127, FOV of 120 mm, and slice thickness of 3 mm. The compression ratio was calculated based on midsagittal MR images using the formula^12^: MRI compression ratio (%) = 1 − (2*c*/(*a* + *b*)) × 100%, where *c* represents the anterior and posterior tube diameter at maximum compression level; *a* refers to the nearest normal level above the compression site; and *b* indicates the nearest normal level below the compression site.

### 2.14 Statistical analysis

The results were analyzed using Prism 5.0, and SPSS software, version 25. The grip strength test was analyzed using one‐way analysis of variance, followed by post hoc tests. Comparisons between decompression and compression groups were performed using two‐tailed *t* tests. Repeated measures data from the experiment were analyzed using linear mixed models and repeated‐measures analysis of variance. The number of animals in each group is provided in both the Results and figure legends. Data are presented as mean ± SD (standard deviation) or box plots, with values considered statistically significant at *p* < 0.05.

## 3 RESULTS

### 3.1 General conditions

**FIGURE 3:**
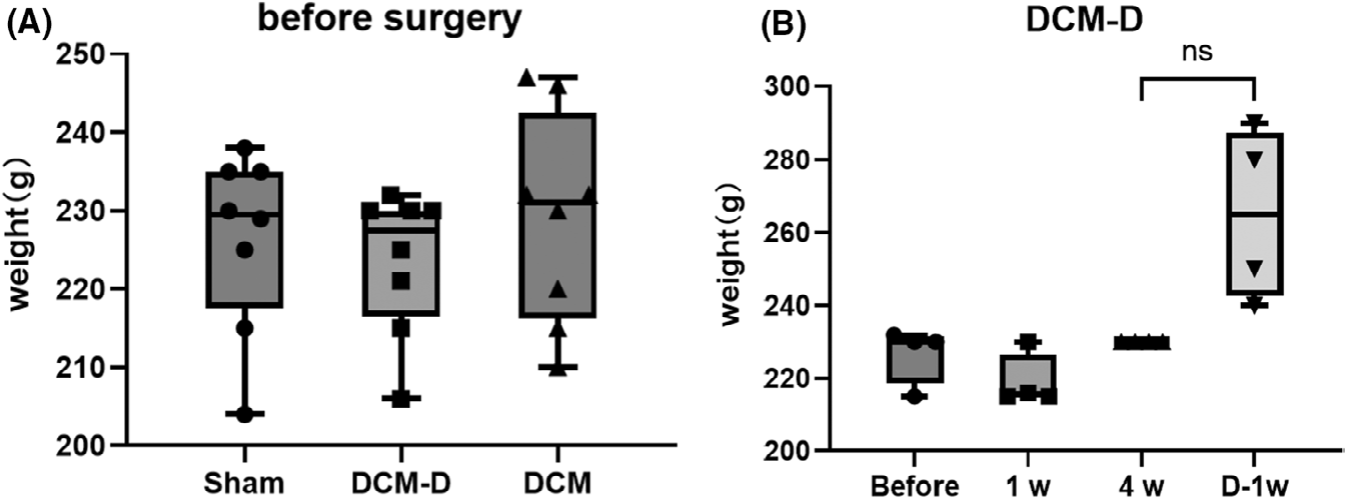
Pre‐experimental rat weight and weight changes during the experiment in DCM‐D group rats. (A) There was no significant change in the weight of the rats before compression across all the three groups. (B) There was no statistical difference in body weight between the DCM‐D group of rats after 4 weeks of compression surgery(4w) and 1week of decompression surgery(D‐1w).

We noticed that one rat each in the DCM and DCM‐D groups exhibited motor complications after compression surgery, including impaired body support and gait patterns and forelimb dysfunction. After decompression, two rats in the sham operation group experienced gait disorder, but were still able to support their body weight. In the DCM group, one additional rat exhibited weakness in the forelimbs and was unable to perform grip strength tests or complete a full gait test. Following decompression, forelimb spasticity was relieved and the limp was reduced in rats from the DCM group; however, one rat continued to experience severe gait disorder and could not complete the gait test (Table 1 and Figure 3).

**TABLE 1:** Motor complications after compression surgery.

Groups	1 Week	4 Weeks	Decompression‐1 week (D‐1w)
Sham	0	0	2 Animals
DCM	1 Animal	0	1 Animal
DCM‐D	1 Animal	0	1 Animal

### 3.2 Surgical decompression has the potential to alleviate forelimb pain response

**FIGURE 4:**
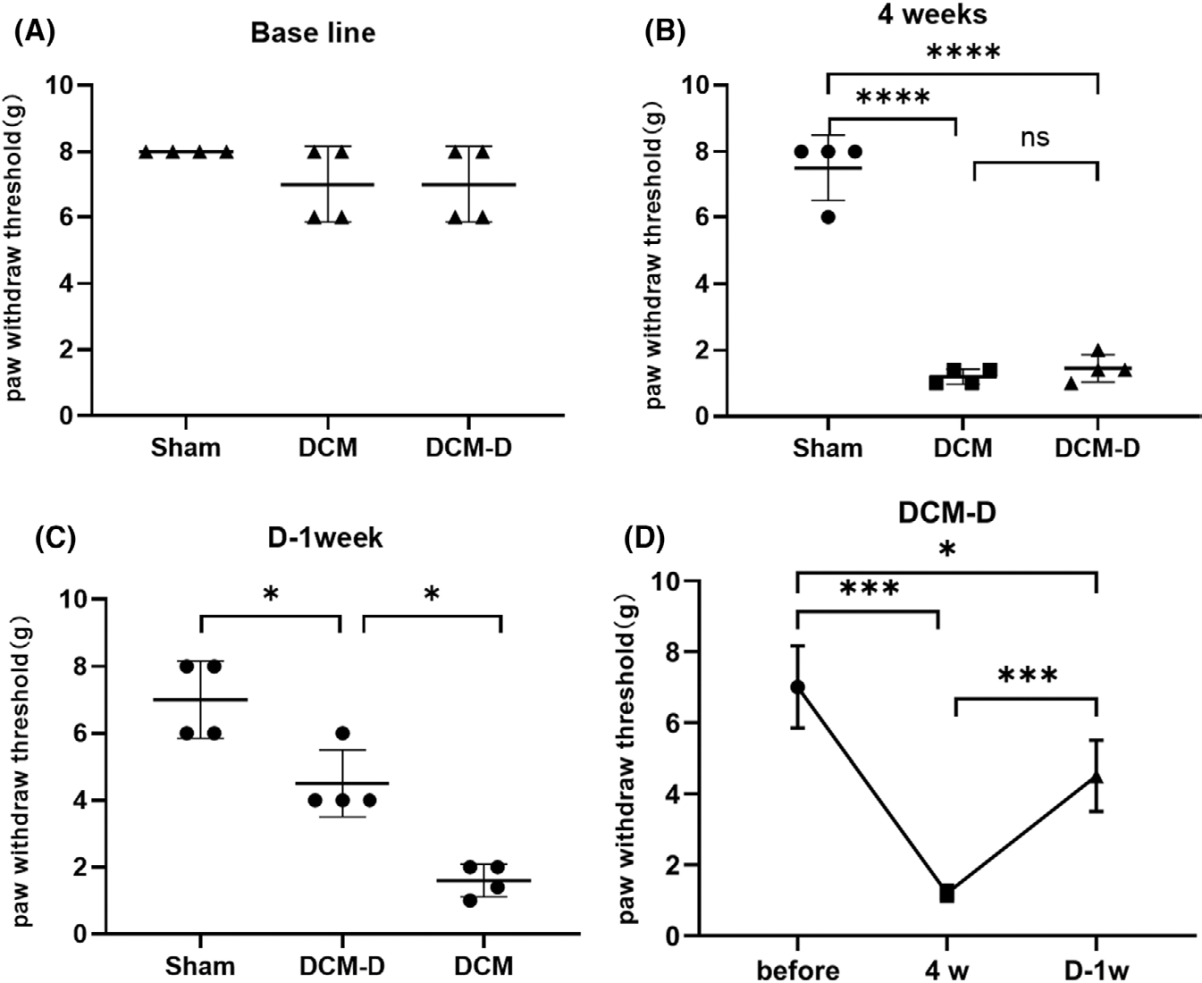
(A) There were no significant differences in baseline data among the three groups. (B) Following 4 weeks of compression, there were statistically significant variations in pain thresholds observed among the three groups (DCM group, DCM‐D group, and sham group), with a *p* < 0.0001 obtained from a two‐tailed *t* test. No significant difference was found between the DCM and DCM‐D groups. (C) One week after completion of decompression surgery, notable disparities in pain thresholds emerged between the sham group and the DCM‐D group, as well as between the DCM‐D group and the DCM group (*p* < 0.05; two‐tailed *t* test; *n* = 4). (D) Within DCM‐D group, assessments before compression and after 4 weeks of compression have significant difference (*p* < 0.001), after 4 weeks of compression and after 1 week of decompression revealed that decompression significantly alleviated pain (*p* < 0.001). These findings were derived using a linear mixed model approach with *n* = 4 participants.

Four weeks after the implantation of the compression material, the pain threshold in the sham group was significantly higher than that in the two compression groups. One week after the decompression procedure, the pain threshold of the DCM‐D group increased significantly, and was significantly different from that of the DCM group but also significantly different from that of the sham group. Among the three time points in the decompression group, the thresholds after decompression were significantly higher than those before decompression but lower than those without compression (Figure 4).

### 3.3 Decompression surgery led to a substantial enhancement in rats' grip strength

**FIGURE 5:**
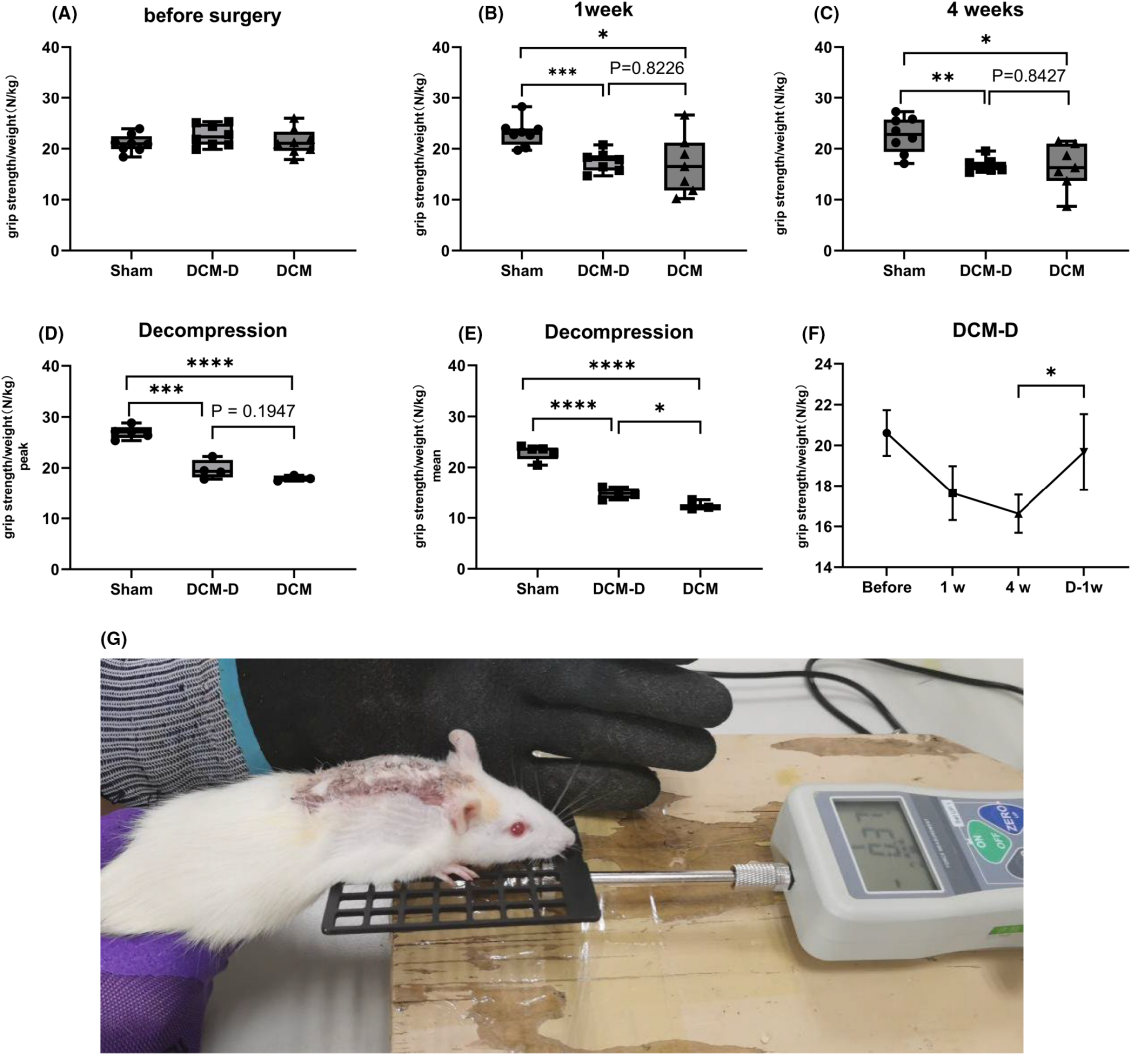
(A) There were no significant differences in standardized grip strength among the three groups before compression. (B, C) A comparison of standardized grip strength was conducted between rats at 1 week and 4 weeks after compression. Significant differences were observed between the sham group and the DCM group at both time points (*p < 0.05). Specifically, at 1 week, there was a highly significant difference (***p < 0.001), while at 4 weeks, the difference remained statistically significant (**p < 0.01). No significant difference was found between the DCM group and the DCM‐D group. The analysis employed a two‐tailed *t* test with a sample size of *n* = 7. (D, E) One week after compression, there were significant differences in peak standardized grip strength (***p < 0.001) and mean standardized grip strength (****p < 0.0001) between the Sham group and DCM‐D group. A significant difference was also observed between the Sham group and the DCM group (****p < 0.0001). No significant difference in peak standardized grip strength was found between the DCM‐D group and the DCM group. Notably, a significant difference in mean standardized grip strength (*p < 0.05) was observed. The statistical analysis employed a two‐tailed *t* test with sample sizes of *n* = 3 for the Sham groups and *n* = 4 for the other groups. (F) In the DCM‐D group, peak standardized grip strength of 4 weeks after decompression decreased compared to 1 week after compression (ANOVA for repeated measures; *n* = 4). (G) Rat grip test diagram.

At 1 and 4 weeks post‐compression surgery, the sham group exhibited significantly higher standardized grip strength compared to the DCM and DCM‐D groups. This suggests that spinal cord compression negatively affects grip strength in rats. One week following decompression surgery, the average grip strength of the DCM‐D group was noticeably higher than that of the DCM group but still lower than that observed in the sham group. Standardized grip strength in the DCM‐D group showed a significant increase 1 week after decompression. These results indicate that decompression surgery restored forelimb strength in rats, but not to levels comparable to those observed in the sham group (Figure 5).

### 3.4 Gait analysis of rats undergoing decompression surgery

**FIGURE 6:**
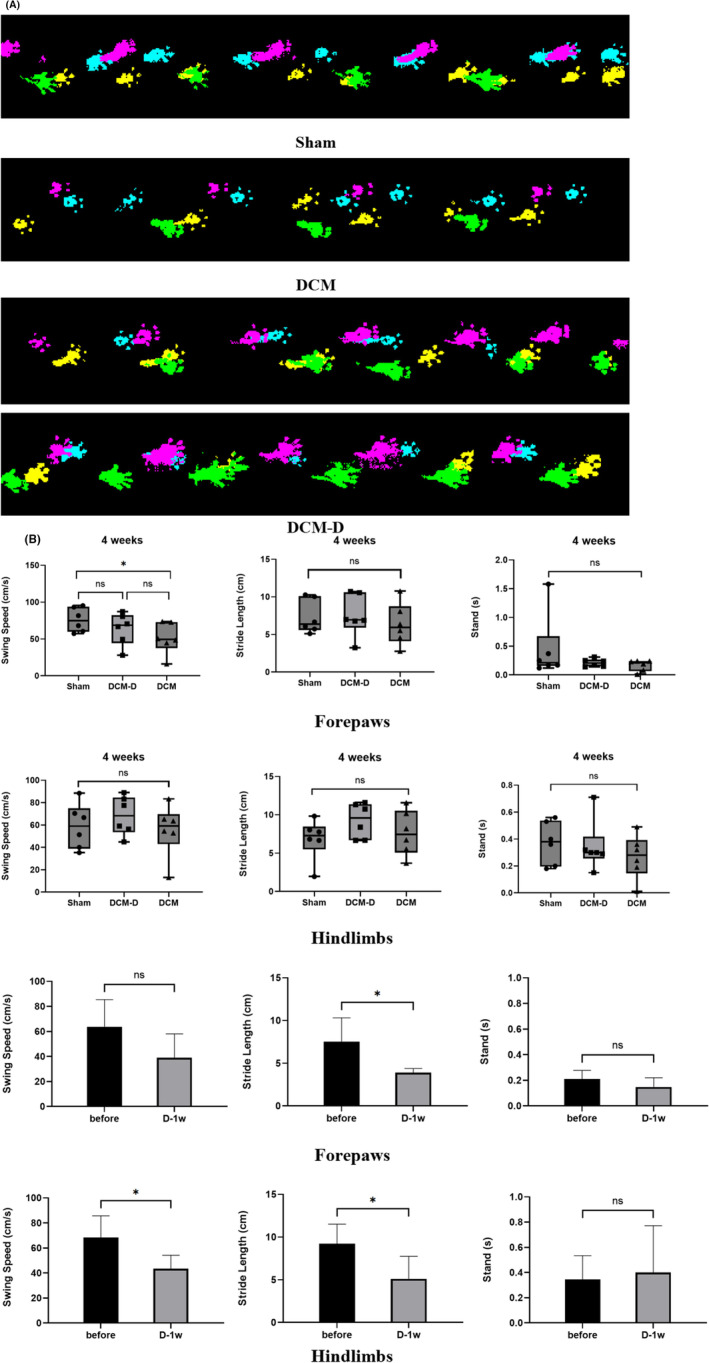
The gait of rats did not improve after decompression. (A) Representative gait images of three groups of rats, blue and purple for right forelimb and right hindlimb, and yellow and green for left forelimb and left hindlimb. The upper and lower panels of the DCM‐D group images show the gait of rats before and after decompression, respectively. (B) After 4 weeks of compression, the swing speed of the forelimbs of the sham group was significantly higher than that of the compression group (*p < 0.05). There was no significant difference in the standing time and stride length of the forelimbs and hindlimbs. In the decompression group, the forelimb stride length after decompression was significantly shorter than that before decompression (*p < 0.05), and the hindlimb swing speed and stride length after decompression were significantly lower than those before decompression (*p < 0.05). There was no significant difference in other gait parameters. Two‐tailed *t* test, *n* = 4.

Before surgery, DCM animals exhibited significant differences from the sham‐operated group solely during the standing phase, while no notable disparities were detected in swing speed and stride length. Upon comparing the pre‐ and post‐decompression stages, a decrease in stride length after decompression surgery was observed in both the fore‐ and hindlimbs of the rats, with the hindlimb swing speed being lower than that before decompression. However, no substantial improvement was noted in other gait parameters, suggesting that decompression surgery did not effectively alleviate the gait disorders in DCM rats (Figure 6).

### 3.5 Blood perfusion of the spinal cord was observed to commence within a time frame of 10 min after decompression

**FIGURE 7:**
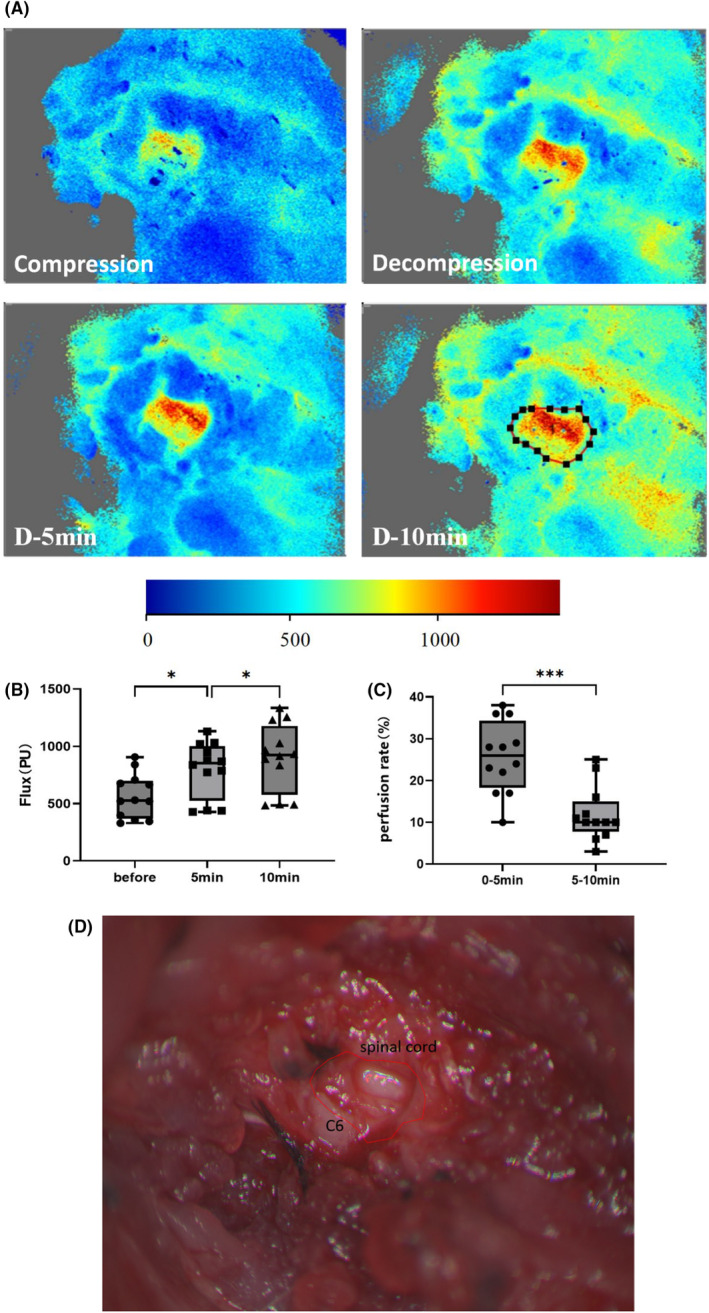
Spinal cord blood perfusion gradually increased within 10 min of decompression. (A) Blood perfusion images of rats were captured at four time points: Before decompression, immediately after decompression, 5 min after decompression, and 10 min after decompression. The region of interest (ROI) highlighted in the figure corresponds to the dorsal spinal cord beneath the exposed C6 lamina. Brighter colors indicate higher blood perfusion. (B) Statistical analysis revealed that the average blood perfusion in this region was significantly greater 5 min after decompression than that before decompression (one‐way ANOVA, *n* = 4). (C) Furthermore, there was a significant increase in perfusion at 10 min after decompression compared to that observed at 5 min post‐decompression. Additionally, the rate of perfusion during the initial 0–5 min period was higher than that during the subsequent 5–10 min interval (two‐tailed *t* test, *n* = 4). (D) Anatomy of the cervical spine and spinal cord of rats during blood flow testing.

The blood flow measurement results demonstrated a gradual increase in the blood flow of rats at three time points: before decompression and at 5 and 10 min after decompression. This finding provides evidence that blood flow reperfusion occurs within the spinal cord of rats within 10 min of decompression. The perfusion volume within the first 5 min of decompression was determined by subtracting the blood flow 5 min after decompression from that before decompression, and that within 5–10 min of decompression was calculated using a similar approach. The ratio between these two perfusion volumes and the blood flow measured 10 min after decompression served as an indicator for assessing the perfusion rate during each respective 5‐min period. Notably, a significantly higher perfusion rate was observed during the initial 0–5 minute period than during the subsequent 5–10 minute period post‐decompression. These findings highlight a faster restoration of blood flow within the first 5 min following decompressive intervention than between 5 and 10 min post‐intervention (Figure 7).

### 3.6 HE staining

**FIGURE 8:**
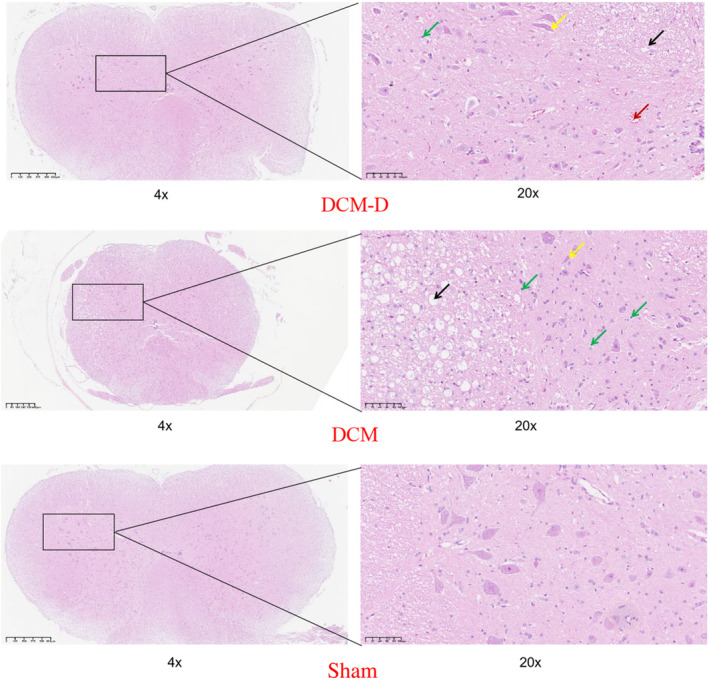
HE staining of compressed segments in three groups of rats. Yellow arrow, neuron nuclear condensation; green arrow, glial cell; black arrow, vacuolated cell; red arrow, vascular congestion.

In the DCM group, the delineation of gray and white matter in the spinal cord was indistinct, with significant atrophy of gray matter, a notable reduction in neuron count, condensation and diminishment of numerous nuclei, as well as pronounced proliferation of glial cells. In the DCM‐D group, there was clearer demarcation between gray and white matter; although there was a slight reduction in neuronal count within the gray matter and some condensed or diminished nuclei were observed, blood vessels appeared dilated and congested, with limited glial cell proliferation. Additionally, a few slightly vacuolated cells were evident in the white matter with more uniform arrangement. Conversely, in the sham‐operated group, spinal cord tissue structure remained intact with clear demarcation between gray and white matter; neuronal cell nuclei exhibited large and evenly distributed characteristics without any discernible histopathological changes (Figure 8).

### 3.7 Enhanced expression of 8‐oxoG DNA in the dorsal horn of the rat spinal cord following decompression

**FIGURE 9:**
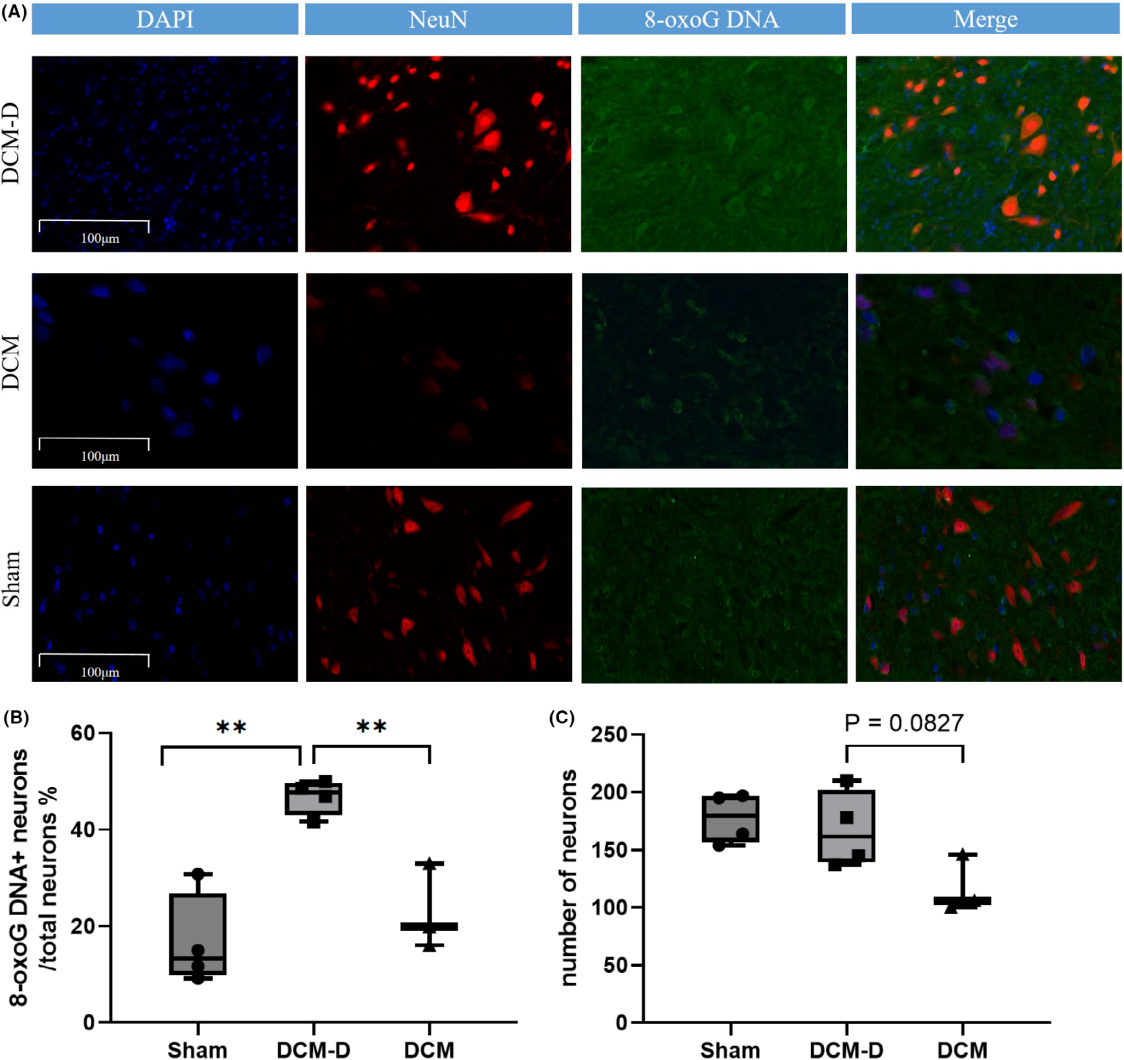
(A) Representative confocal images of rat cervical spinal cord sections stained with 8‐oxoG DNA and NeuN are shown (scale bar, 100 μm). (B) At 1 week post‐decompression (5 weeks after compression surgery), the proportion of neurons exhibiting 8‐oxoG DNA was significantly increased in the decompression group compared to the Sham group. Each group consisted of *n* = 3,4 animals. Statistical analysis revealed a highly significant difference (**p < 0.001) using one‐way ANOVA followed by Tukey's post hoc test. (C) The number of NeuN‐positive cells in the dorsal horn of the spinal cord at 1 week post‐decompression did not show any significant differences between the DCM‐D, DCM, and Sham groups.

After a week of decompression, there was no statistically significant difference in the number of NeuNs in the dorsal horn of the spinal cord among rats in the DCM‐D group compared to the other two groups. However, it was found that higher expression of 8‐oxoG DNA occurred within the NeuNs of rats belonging to the DCM‐D group, as opposed to those in the other two groups. This finding suggests that oxidative damage was inflicted on neurons in rats in the DCM‐D group, thereby providing evidence of ischemia–reperfusion injury occurring within rat neurons after decompression (Figure 9).

### 3.8 Compression ratio

**FIGURE 10:**
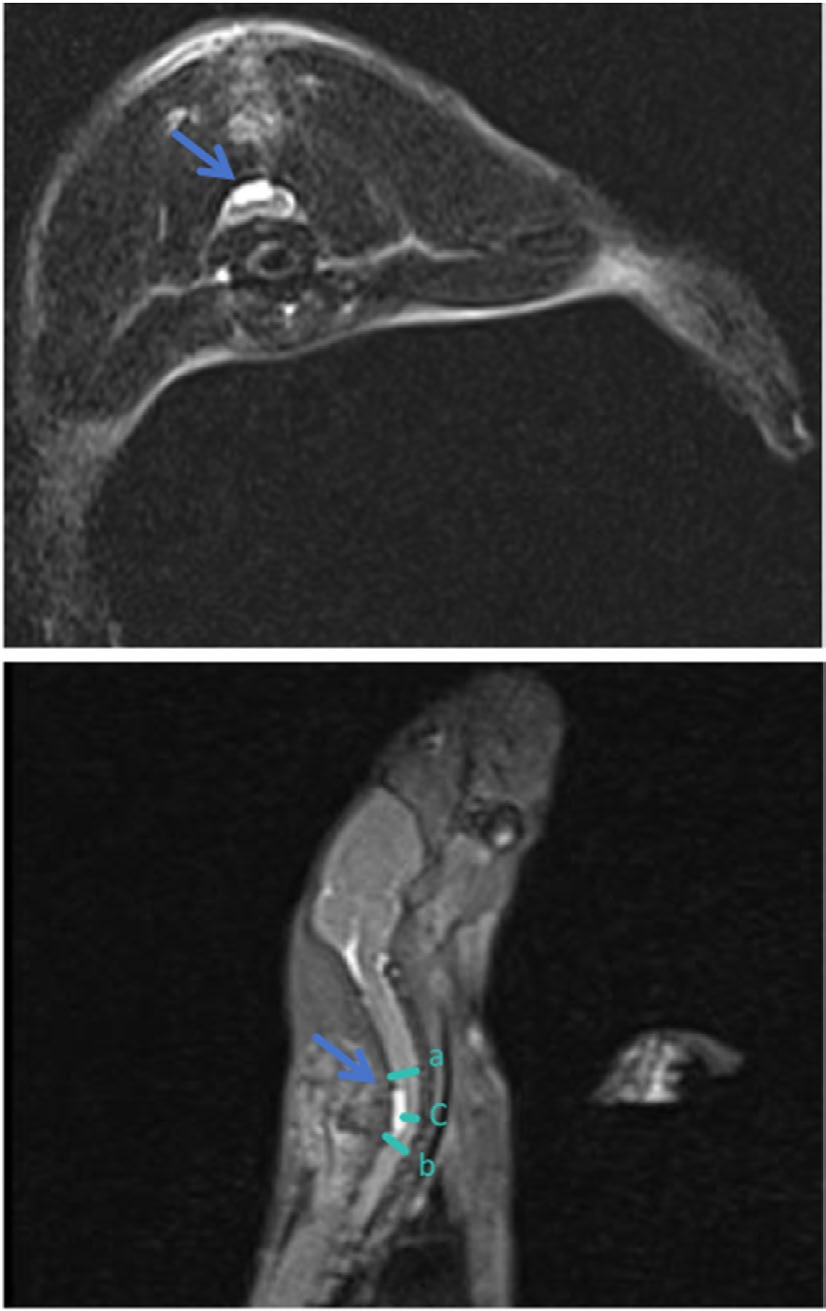
The sagittal and transverse MRI images of DCM rats depict the presence of compression material, indicated by the arrow within a white square in the spinal canal. This material is located beneath the C6‐7 lamina, situated on the posterior aspect of the spinal cord. The three line segments a, b, and c in the figure represent the diameter of the spinal canal, which is employed in the calculation of the spinal cord compression rate. Compression rate is quantified to be 53.8%.

The spinal MRI images of the rats are presented in Figure 10, illustrating a calculated compression rate of 53.8% within the spinal canal. This figure shows the presence of compression material beneath the C6‐7 lamina in the rat model. Transverse imaging revealed that the material was centrally located within the spinal canal and remained structurally intact without any signs of damage. Additionally, signal abnormalities indicative of spinal cord ischemia were observed within the compressed segment. These findings provide compelling evidence that apart from mechanical compression of the spinal cord, such as edema or mechanical injury to the nerve roots or laminae, acute injuries are not induced by the compression material (Figure 10).

## 4 DISCUSSION

In this study, we successfully developed a rat model of chronic cervical spinal cord compression and decompression that accurately replicates the spatial and temporal characteristics of the corresponding human disease. The feasibility of spinal cord decompression and surgical decompression was confirmed using MRI analysis. Notably, rats subjected to spinal cord compression and subsequent decompression exhibited significant gait patterns and forelimb dysfunction, closely resembling those observed in humans with DCM who underwent decompression. The rat spinal cord exhibited increased local blood flow after decompression surgery, indicating the occurrence of ischemia–reperfusion. Notably, post‐surgical neuronal oxidative damage induced by SCII has been observed in rats. This comprehensive animal model provides valuable insights into superficial spinal cord blood flow dynamics, histological alterations within the spinal cord tissue, neuronal oxidative damage mechanisms, and neuropathic pain associated with this condition.

Blood supply characteristics of the spinal cord in rats are comparable to those observed in humans,^13^ where 8–12 root arteries converge into the anterior spinal artery, accompanied by two posterior spinal arteries. Spinal cord ischemia, resulting from compression, plays a crucial role in the pathological progression of DCM. Karadimas et al.^8^ utilized a rat model of spinal cord compression and observed a reduction in capillary density and cross‐sectional area within the gray and white matter of the compressed segment. This study provides evidence that mechanical compression can induce ischemia in the affected region of spinal cord microcirculation. Lebret et al.^14^ employed intravoxel incoherent motion imaging (IVIM) to ascertain that individuals with DCM exhibited lower blood flow velocity (BV) and blood flow (BF) index in the C1–C3 segments compared to healthy subjects. The reduction in microvascular volume fraction was found to be associated with spinal cord gray matter atrophy, thereby confirming that compression leads to hemodynamic alterations above the site of cervical cord compression in DCM patients. Furthermore, this study demonstrated a correlation between compression‐induced changes in blood perfusion and the underlying neurodegenerative process, thus providing evidence for the interplay between vascular dynamics and neurological degeneration. In the current study, limited techniques existed for detecting alterations in blood perfusion before and after spinal cord compression in rats. Karadimas et al.^10^ employed the fluid‐sensitive alternating inversion recovery (FAIR) technique of magnetic resonance imaging (MRI) to assess the spinal cord blood flow. Their findings revealed a decrease in spinal cord blood flow 6 weeks after implantation compared to measurements taken within the same area 24 h after decompression surgery. Vidal et al.^15^ employed power Doppler to quantify the number of functional blood vessels before and after decompression, revealing a higher count at 2 weeks after decompression than at 24 h post‐decompression. In the mouse model of Vidal et al.,^8^ the technique of assessing spinal cord blood flow using fluorescent particles injected into the mouse heart demonstrated greater fluorescence absorbance in the decompression group than that in the compression group. The above studies are influenced by contrast media and individual differences and lack direct evidence of specific changes in blood flow. Laser speckle contrast imaging (LSCI) is a real‐time imaging tool, enabling the real‐time visualization of surface‐level blood flow in rat tissues.^16^ LSCI commonly utilizes a low‐intensity near‐infrared laser, a low‐power and long‐wavelength light source, to irradiate spinal. The light is then directed towards the device sensor to gather data on blood flow dynamics, which is subsequently captured by a camera. The resulting speckle pattern in the image is further analyzed using computer software to quantify pixel movement. In this study, a LSCI system was used to investigate the blood flow reperfusion process in the rat spinal cord following decompression. The change in blood perfusion within 0–5 min after decompression was greater than that observed within 5–10 min after decompression, indicating a transition from rapid to gradual restoration of blood perfusion. Previous studies have demonstrated that alterations in vascular morphology play a crucial role in modulating spinal cord blood flow in individuals with DCM, and it takes some time to establish compensatory vascular growth and an adequate blood supply. During the early stages of decompression, alleviation of mechanical compression of blood vessels and recovery of vascular morphology may primarily contribute to microcirculation perfusion.

DCM can arise from both static and dynamic pressure. Among these factors, thickening of the ligamentum flavum is a crucial pathological element.^17^ In recent studies, animal models of DCM have primarily aimed to simulate the process of ligamentum flavum hypertrophy. In this context, material selection and postoperative complications are significant challenges encountered by these models. Currently, the materials utilized for spinal cord compression predominantly include balloons, water‐absorbent expansion materials, and synthetic aromatic polyether materials.^15^, ^18^, ^19^ The water‐absorbing expansile material employed in this study was capable of absorbing interstitial fluid expansion in the rats and attained its maximum potential within a time frame of 48–72 hours.^20^ Furthermore, it has been verified that this material does not induce adverse reactions in rat tissues.^21^ The advantages of the water‐absorbing expansion material include (1) uniform expansion and compression of the spinal cord; (2) minimal structural damage to the spinal canal, limited to mechanical compression only, resulting in low infection rates and high animal survival rates; (3) a straightforward procedure that eliminates the need for artificial expansion. Some scholars argue that the modeling method utilizing a water‐absorbent expansion material represents a subacute compression model,^22^ as it exhibits short‐term expansion without reflecting the progressive compression observed in DCM progression, but rather simulates continuous compression processes. The size of the materials used in this study was validated through preliminary experiments. It induces a specific occupation within the spinal canal, resulting in the mechanical compression of the spinal cord. In the sham group, the material was compressed for 5 min and subsequently removed. No infectious postoperative complications were found in the sham group within 4 weeks of pressurization. Rats in both the DCM and DCM‐D groups exhibited relatively stable spinal cord compression without acute spinal cord contusion, thereby confirming that the requirements for spinal cord compression were met in this study. The rat models were closely monitored for complications following surgery and treated accordingly. To enhance the success rate of the model, we narrowed down the exposed incision (C4‐T2) and reduced the operation time. Previous methods for DCM decompression predominantly involved full laminectomy of the compression segment.^8^, ^9^, ^10^, ^15^ However, studies involving this operation method did not provide information on the internal fixation measures of the rat cervical spine after laminectomy, thereby leaving room for potential mechanical structural instability in the postoperative phase. To mitigate confounding factors, unilateral laminectomy was performed to achieve decompression.

Behavioral tests were conducted in rats based on typical clinical presentations observed in patients with DCM, such as paresthesia and hypokinesia. Notably, upper limb pain, weakness, and gait disturbances are common observed manifestations.^1^, ^23^ Additionally, persistent hyperalgesia is a characteristic symptom of spinal cord injury.^24^ The present study indicate that hyperalgesia persisted after decompression in rats, whereas no changes were observed in the sham operation group, suggesting that this phenomenon is unrelated to spinal cord contusions or other confounding factors. Decreased upper‐limb strength and gait disturbance are prevalent symptoms in patients diagnosed with DCM.^25^, ^26^ A study conducted by Ram et al. revealed that patients diagnosed with DCM exhibited a significant reduction in walking speed, slower pace, longer stride length, and wider step width than their healthy counterparts. Notably, the gait parameters of patients showed improvement following surgery.^27^ In this study involving DCM rats, it was observed that they walked at a slower pace than the rats in the sham group. Despite decompression procedures being performed on the rats, no improvement in speed or stride length was observed; instead, a decrease in stride length was noted. This phenomenon may be attributed to postoperative SCII. Meanwhile, both DCM and decompressive animals exhibited impaired ambulation throughout the experiment, necessitating multiple attempts for the rats to complete the gait analysis test. Some DCM rats were unable to successfully undergo gait analysis, rendering their data unsuitable for inclusion in our analysis. This discrepancy may partially account for the incongruity observed between our findings and clinical research outcomes. Grip strength serves as a pivotal indicator for assessing upper limb functionality in individuals with DCM, with diminished hand strength representing a characteristic manifestation in these patients.^28^, ^29^ Maximum grip strength serves as a convenient and reliable measure for assessing forelimb strength, whereas average grip strength encompasses the evaluation of muscle fatigue.^28^, ^30^ Notably, individuals with cervical spinal cord injury exhibit a higher muscle fatigue index than healthy individuals.^31^ The handgrip strength meter used in this study was a digital isometric dynamometer that enabled real‐time monitoring of grip strength variations in rats. Throughout the measurement process, the hindlimbs of the rats were immobilized while recording the axial strength exerted by their forelimbs. The findings revealed that DCM rats exhibited lower standardized peak grip strength than the sham group, whereas the average grip strength post‐decompression surpassed that observed before surgery. These findings suggest that post‐decompression muscle strength in rats exhibits greater fatigue resistance than pre‐decompression levels, implying a potential reduction in cervical spinal cord injury through decompression surgery.

Previous studies have documented various tissue alterations in the spinal cord of patients with DCM, including spinal cord flattening, scar tissue hyperplasia, central gray and white matter degeneration, demyelination in the posterolateral and anterolateral columns, and neuronal loss in the anterior horn.^8^, ^23^ Dhillon et al. successfully established rat models of DCM and performed decompression surgery. Their investigation revealed axonal degeneration and neuronal apoptosis in the DCM group; however, surgical decompression effectively mitigated ongoing cell loss by suppressing apoptosis.^32^ SCII induces oxidative stress and the excessive generation and accumulation of reactive oxygen species (ROS).^33^ ROS‐induced DNA damage can result in impaired cellular function, including apoptosis or necrosis, while simultaneously activating the DNA damage response and repair (DDR/R) network. Among various oxidative lesions, 8‐oxoG DNA is the most prevalent form of oxidative damage (DDR/R). Furthermore, chronic inflammation, cancer, premature aging, and certain tumor‐like diseases can also contribute to the production of 8‐oxoG DNA.^34^, ^35^, ^36^ No significant alterations were observed in the number of neurons following decompression surgery in the DCM‐D group. However, a higher proportion of neurons exhibited 8‐oxoG DNA retention than in both the DCM and sham‐operated groups, indicating the occurrence of spinal cord ischemia–reperfusion injury after decompression. Previous investigations have demonstrated that the DNA damage response and repair (DDR/R) network can effectively detect and rectify DNA damage, thereby enhancing an organism's capacity to counteract oxidative stress‐induced damage triggered by endogenous or exogenous stimuli.^37^, ^38^ The production of 8‐oxoG DNA may also arise from immune cell dysfunction or the secretion of a substantial quantity of inflammatory factors.^39^, ^40^ In the pathological progression of DCM and decompression surgery, a chronic inflammatory response is one of the pivotal mechanisms,^21^, ^41^ leading to the generation of 8‐oxoG DNA in neurons within DCM rats. In this study, the rats in both the DCM and sham groups underwent identical surgical procedures during decompression surgery, resulting in immune system activation due to trauma across all three groups. The DCM‐D group exhibited more severe oxidative damage than the other two groups, suggesting the occurrence of SCIII following decompression.

This study has certain limitations. The gait data from rats with severe symptoms of DCM may exhibit significant differences compared to those of rats with mild symptoms. However, due to our inability to obtain complete gait data from some rats with severe DCM symptoms, we were unable to demonstrate a difference in gait. In this context, we suggest that complete gait data from DCM rats could be more easily obtained by using stimuli such as food induction. Furthermore, it should be noted that laser speckle contrast imaging has limited detection depth and may be influenced by changes in blood flow within the spinal dura mater.

## 5 CONCLUSIONS

The novel rat spinal cord decompression model described in this study successfully induced neurological deficits and neuropathological features that accurately replicated human conditions. Moreover, it was compatible with MRI, providing evidence that ischemia–reperfusion injury occurred locally in the rat spinal cord following decompression surgery, from a microcirculatory perspective. Consequently, this model serves as an invaluable tool for future studies on spinal cord injury and has the potential to advance our understanding of the mechanisms underlying chronic spinal cord injury.

## AUTHOR CONTRIBUTIONS

**Boyu Zhang:** Data curation; validation; writing – original draft. **Zhefeng Jin:** Funding acquisition; software; supervision; writing – review and editing. **Pengren Luo:** Validation; visualization; writing – original draft. **He Yin:** Resources; software; supervision. **Xin Chen:** Supervision; validation; writing – review and editing. **Bowen Yang:** Data curation; methodology; supervision. **Bo Xu:** Investigation; methodology; resources. **Xiaokuan Qin:** Resources; software; validation. **LiGuo Zhu:** Conceptualization; resources; supervision; writing – review and editing. **Guoliang Ma:** Validation; visualization. **Dian Zhang:** Software; supervision; validation.

## FUNDING INFORMATION

This work was carried out under the Nursery Training Program of the Wangjing Hospital Affiliated to China Academy of Chinese Medical SciencesNursery Training Program of the Wang Jing Hospital of China Academy of Chinese Medical Sciences.

## CONFLICT OF INTEREST STATEMENT

The authors declare that they have no affiliations with or involvement in any organization or entity with any financial interest in the subject matter or materials discussed in this manuscript.

## ETHICS STATEMENT

Our research were approved by Ethics Committee of Beijing Long'an Experimental Animal Breeding Center (approval number: BJLongan‐L‐L‐00015).

## Supporting information




Data S1:

## Data Availability

All relevant data are within the paper and its Supporting Information files.
